# A review on SARS-CoV-2 and stroke pathogenesis and outcome

**DOI:** 10.1186/s41983-021-00319-y

**Published:** 2021-05-19

**Authors:** Tamer Roushdy, Eman Hamid

**Affiliations:** Neurology Department, Faculty of Medicine, Ain Shams University, 38 Abbasia, PO 11591, Cairo, Egypt

**Keywords:** Corona virus, Stroke pathogenesis, Endotheliopathy, Angiotensin-converting enzyme 2, COVID-19

## Abstract

Severe acute respiratory syndrome corona virus 2 hit strongly and hardly the entire globe for more than 1 year with a morbidity exceeding 139 million and a mortality approaching 3 million worldwide since its emergence in China in December 2019 until April 2021.

Although being termed after its ancestor the acute respiratory syndrome corona virus that emerged in 2002. Yet, the current corona virus has its unique devastating presentations being pulmonary and extra pulmonary.

In the current review, a highlight on the role played by corona virus 2 on pathogenesis and outcome of stroke is presented with an attempt to point to the most approved ways through which the corona virus induce stroke being disturbance in renin angiotensin system and angiotensin-converting enzyme 2 receptors downregulation, endothelial cell damage with coagulopathy, cytokine storm, and platelet as well as outcome and risks in patients who are suffering stroke with modifiable vascular risk factors and catching the severe acute respiratory syndrome corona virus 2.

## Introduction

In December 2019, a severe acute lower respiratory tract syndrome was detected and declared an epidemic in Wuhan City of China that was later on returned to a novel severe acute respiratory syndrome corona virus 2 (SARS-CoV-2).

SARS-CoV-2 was globally responsible for the corona virus disease 2019 (COVID-19) pandemic that was declared by the world health organization in March 2020.

Coronaviruses are well known since the outbreak of severe acute respiratory syndrome corona virus (SARS-CoV) in the year 2002 in China, and the Middle East respiratory syndrome corona virus (MERS-CoV) in the year 2012 [1, 2]. Yet, what makes SARS-CoV-2 a virus of interest for research and reviews is its great impact on health providing services as well as on economy worldwide and its ongoing mutation that questions the possibility of containing it. Besides that SARS-CoV-2 manifestations does not stand at respiratory system only but reaches other systems with cerebrovascular one of them.

SARS-CoV-2 is composed of a spike (S)-shaped protein emerging from a lipid envelope. S protein binding to angiotensin-converting enzyme 2 receptor (ACE 2) is the way through which the virus gains entrance to cells [3].

Pathogenesis and pathophysiology of stroke in SARS-CoV-2 is believed to be multifactorial. The following review aims at discussing the commonest pathophysiological associations between SARS-CoV-2 and stroke being disturbance in renin angiotensin system and angiotensin-converting enzyme 2 receptors downregulation, endothelial cell damage with coagulopathy, cytokine storm, and platelets as well as highlighting prognosis of stroke with COVID-19.

## Main text

### Pathogenesis of stroke with SARS-CoV-2

#### Renin angiotensin system (RAS) and angiotensin-converting enzyme 2 receptor (ACE 2)

ACE 2 receptors are present within different organs in the human body such as the lungs, the heart, the small bowel, the kidneys, the blood vessels, and the brain. Precisely ACE 2 receptors are abundant along epithelial and endothelial cells of different body organs [4].

ACE 2 receptors play an important role in the renin-angiotensin-system (RAS) and any disruption in their state either by downregulation or upregulation may have an impact on RAS that in turn affects modifiable vascular risk factors as hypertension and increases the risk for stroke [5].

**Fig. 1:**
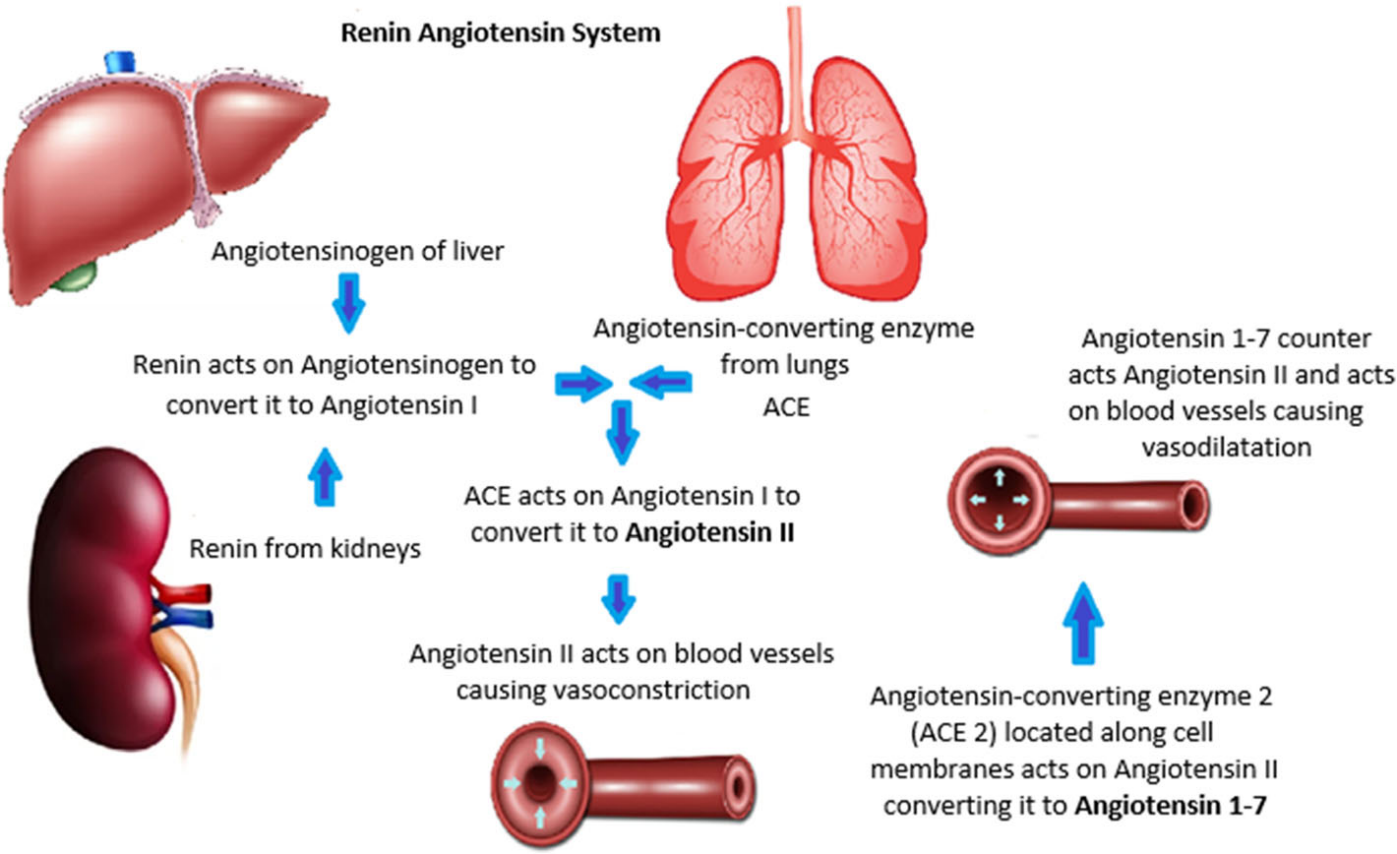
Renin angiotensin system (RAS)

RAS regulates blood pressure through its production of a potent vasoconstrictor which is angiotensin II that is the result of action of renin on angiotensinogen converting it to angiotensin I and through angiotensin-converting enzyme (ACE) angiotensin I is metabolized to angiotensin II, after its action is achieved and to prevent excessive rise in blood pressure angiotensin II is degraded by angiotensin-converting enzyme 2 (ACE 2) to the vasodilator angiotensin 1–7 that has an antihypertensive effect [6] (Fig. 1).

ACE 2 are not specific to angiotensin II but they act as well as on bradykinins and prostaglandins [7, 8]. Downregulation of such receptors after being attached to the S protein of SARS-CoV-2 increases angiotensin II with excessive rise in blood pressure that can be attributable for both ischemic and hemorrhagic strokes development and increased bradykinins causes inflammatory states in the lungs with hypoxemia that may further complicate cerebrovascular insults also increase in angiotensin II production increases vascular permeability to the degree of causing pulmonary edema [9].

Although downregulation in ACE 2 with COVID-19 is reported in many studies and was previously confirmed in 2002 with SARS-CoV as well [10] yet, it is questionable by other studies that states that ACE 2 are still functioning despite being attached to the S protein of SARS-CoV-2, questioning the role of RAS dysregulated mechanism in stroke and COVID-19 [11] and that cytokine surge secondary to the virus infection may over express ACE 2 rather than down regulate it and this facilitates more viral entry to cells [12].

So, it appears that ACE 2 expression whether downregulated can cause further hypoxemia, excessive blood pressure rise and volume overload through unopposed angiotensin II action that may play a role in stroke pathogenesis and on the other hand upregulation facilities more viral load that may play a role in stroke pathogenesis through other paths as will be discussed in the next sections.

### Endothelial cells and coagulopathy

Endothelial cells infected with SARS-CoV-2 whether in the lungs, or those lining blood vessels or the heart may have a major role in the pathology of strokes associated with COVID-19 through endotheliopathy [13].

SARS-CoV-2 entry into endothelial cells causes both dysfunction and disruption. Such effects enhance coagulopathy through reducing the action of thrombomodulin which in normal states reduce coagulation [14].

Plasminogen activator inhibitor 1 (PAI-1) is elevated in patients with COVID-19. Since it is mainly produced by endothelial cells [15], its increased level in circulation donates endothelial disruption with SAR-CoV-2. PAI-1 blocks the action of proteins that causes clot lysis so as to reduce possibility of bleeding in injuries. Its increased level in COVID-19 enhances coagulopathy.

Von Willebrand factor (VWF) is another blood component that is responsible for enhancing platelet adherence and stabilizing blood clots is found to be elevated in COVID-19, and with endothelial damage; platelet and thrombus formation within circulation is explainable whether along arterial or venous systems [16, 17].

COVID-19-induced endotheliopathy may weaken the vessel wall causing its rupture which explains hemorrhagic strokes. On the other hand, hemorrhagic transformation is reported in some case series owing to coagulation consumption (CC) yet, CC is rarely found with COVID-19-induced coagulopathy (CIC) where prothrombin time and activated partial thromboplastin time are usually within normal ranges [18].

Meanwhile platelets aggregation on the damaged endothelium causes thrombosis. Damage to the endothelium of the heart also may enhance intracardiac thrombus formation that my further detach causing distal embolization and myocarditis with arrhythmia may aid in distal embolization [19, 20].

**Fig. 2:**
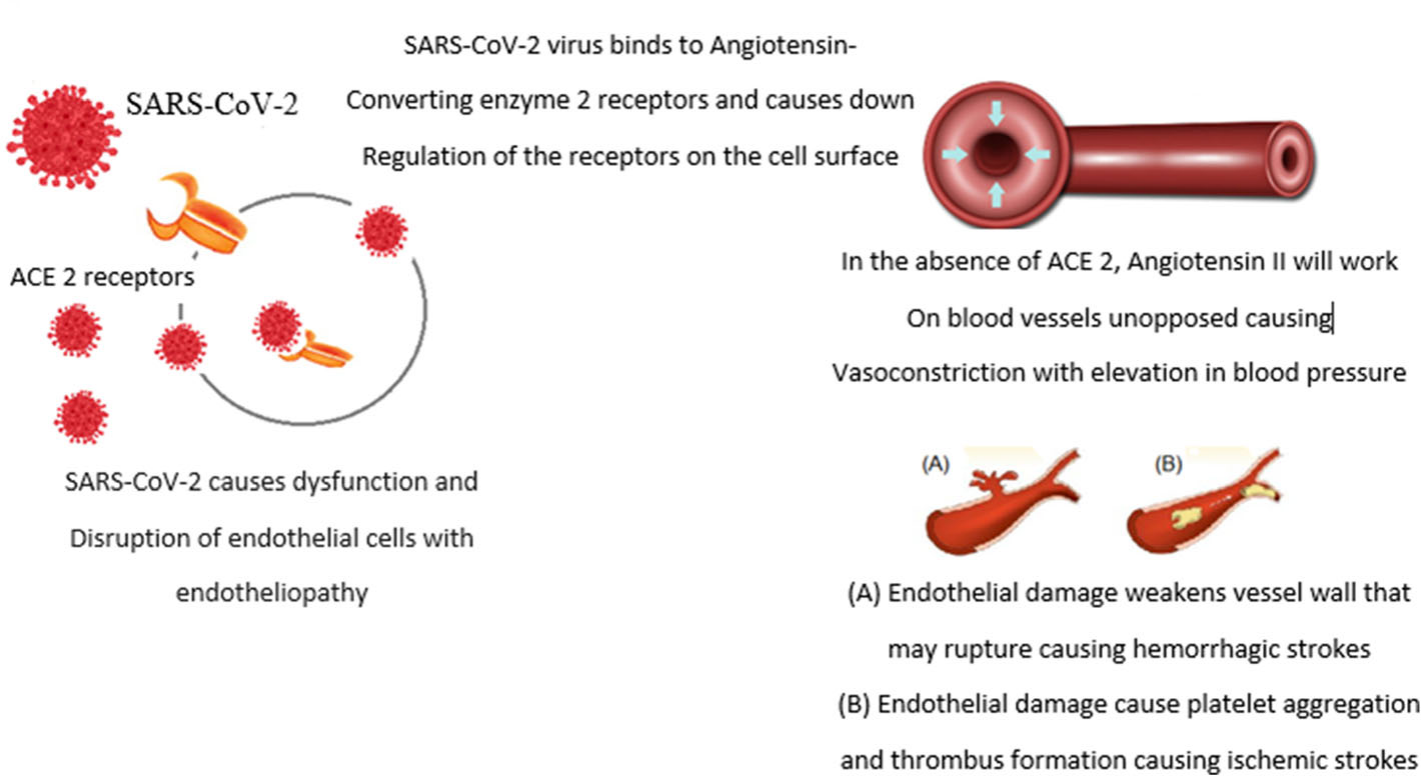
SARS-CoV 2 effect on endothelial cell and vessel wall. SARS-CoV-2 entrance to cells through ACE 2 receptors with down regulation of ACE 2 receptors and on replication and emergence from cells endothelial damage takes place with either vessel wall rupture or vessel lumen narrowing up to occlusion by thrombus, unopposed angiotensin II causes elevation in blood pressure that may cause hemorrhagic or ischemic strokes

So, different etiologies of strokes are likely to occur with SARS-CoV-2 whether hemorrhagic, or ischemic that may further be cardio embolic with a cardiac source or arterial source or thrombotic [21] (Fig. 2).

### Cytokine storm, inflammation, and platelets

Inflammation induced by SARS-CoV-2 enhances platelets to release its inorganic phosphate residues that are termed polyphosphates (poly P). In normal conditions, Poly P stabilizes clot until risk of bleeding from any injury is minimal. In inflammation excessive production of Poly P occurs that causes further activation in factor V and XI promoting thicker clot formation [22].

Being a novel virus that was never encountered by the human immune system, on exposure to it a surge of cytokines and chemokines is released such as interleukin 1, 6, and 8 beside other factors which all cause a macrophage activation like syndrome that further enhances coagulopathy, endotheliopathy, and immunothrombosis [23].

### SARS-CoV-2 and outcome of stroke

Stroke is typically encountered along older age groups than young ones. Yet, in a multiple COVID-19 case series of stroke patients undergoing thrombectomy, it was noted that mean age tend to be younger with an average of 59 years compared to 74 which is the average mean age for such procedure in pre COVID-19 era [24–26].

The reason behind tendency of stroke to affect younger age group who caught SARS-CoV-2 is CIC [27] and this makes COVID-19 an independent risk factor for stroke [28].

Studies assessing gender role in outcome of stroke with COVID-19 are scant, yet Haitao and colleagues [29] have assessed prognosis in COVID-19 infection and its relationship to gender and found that males tend to have severe presentation of COVID-19 with poor prognosis than females secondary to ACE 2 expression, inflammatory markers, and cytokine storm levels as well as habits like smoking. Such findings could be a guide to consider that males who suffer from stroke and SARS-CoV-2 will have a poor prognosis.

Modifiable vascular risk factors such as hypertension, dyslipidemia, and diabetes are associated with pathological downregulation in ACE 2 [30] and such downregulation is further augmented with SARS-CoV-2.

COVID-19 downregulation of ACE 2 [10, 12, 31] leads to excessive production of angiotensin II with loss of its breakdown to the vasodilator metabolite angiotensin 1–7, and this excessive angiotensin II have been responsible for endothelial damage and dysfunction as well as oxidative stress and enhances thrombosis that may be one of the possible explanations of stroke occurrence as well as guarded prognosis [32].

Diabetes is a comorbidity with stroke worldwide as well as in Egypt [33, 34]. Diabetics usually suffer from impaired innate immune response and over expressed cytokines. This impaired immunity in diabetics who are suffering from strokes as well may further intensify SARS-CoV-deleterious effect on stroke outcome [35].

Interleukin 6 was reported by Aref and colleagues [36] to be of prognostic role in ischemic stroke recurrence as well as in mortality. Taking into consideration its surge in cytokine storm induced by COVID-19 and its role in activating other cytokines may further play a role in poor prognosis in stroke victims with superimposed SARS-CoV-2 [37].

Angiotensin-converting enzyme inhibitors and angiotensin II receptor blockers which are commonly prescribed in hypertensive and diabetic patients [38] were accused in some reports of having a drastic effect on COVID-19 victims [39, 40] as by inhibiting the action of ACE an upregulation in ACE 2 occurs which facilitate more SARS-CoV-2 entrance to cells with further replication and intensified symptoms yet this claim was countered by other reports on some animal models [41].

Statins that are used either in the acute phase of stroke as anti-inflammatory medications or in dyslipidemia as a lipid-lowering agents cause upregulation in ACE 2 that facilitates more SARS-CoV-2 entrance to cells with more viral load for patients with dyslipidemia or those who are already stroke sufferers [42].

Smoking by itself is hazardous and is directly linked to stroke development. Chemicals within cigarettes reduce levels of high-density lipoprotein and increase levels of low-density lipoprotein which results in dyslipidemia, atherosclerosis, and stroke development [43].

Nicotine as a chemical in cigarettes increases heart rate and elevates blood pressure and this also can be a reason behind stroke in smokers [43].

The behavior behind smoking which involves frequent hand to mouth contact or sharing smoking-related devices like water pipe, cigarettes, and nicotine-free vapes may play a role in increasing risk of spreading and catching SARS-CoV-2 [44, 45].

Studies on effect of smoking on ACE-2 receptors have a conflicting results. Some stated that smoking reduces ACE-2 receptors [46, 47] while other studies concluded that smoking causes an upregulation in ACE-2 receptors [48, 49]. Yet, both actions have a negative effect on smokers who are exposed to SARS-CoV-2; reduction of ACE-2 receptors will cause an uninterrupted angiotensin II action with elevated blood pressure and endothelial damage. While upregulation in ACE-2 receptors will increase viral entrance to endothelial cells and increases risk of COVID-19.

Outcome of stroke with COVID-19 is guarded since the most common modifiable vascular risk factors being hypertension, diabetes, and dyslipidemia have an effect on as well as being affected by the virus and the ACE 2 receptors by which the virus gain entrance into the cells.

### Limitations and recommendations

COVID-19 is still a novel virus with ongoing mutations. Its role in stroke pathogenesis although have solid scientific basis through different researches yet ought to be replicated on animal models to reach a final agreement on the most common mechanism of action through which it induces stroke.

From the outlined mechanisms through which COVID-19 plays on stroke pathogenesis, it is justifiable to use anticoagulants as well as corticosteroids with SARS-CoV-2 yet dosage and time of initiation still needs further studies.

## Conclusion

SARS-CoV-2 pandemic that has been sieging the globe along an entire year ought to be considered an etiological cause of stroke secondary to associated pathophysiology in the form of ACE 2 downregulation, endotheliopathy, coagulopathy, and associated cytokine storm which all share in pathogenesis of stroke.

## Data Availability

The corresponding author takes full responsibility for the data, has full access to all of the data, and has the right to publish any and all data separate and apart from any sponsor.

## Abbreviations

SARS-CoV-2: Severe acute respiratory syndrome corona virus 2
COVID-19: Corona virus disease 2019
SARS-CoV: Severe acute respiratory syndrome corona virus
MERS-CoV: Middle East respiratory syndrome corona virus
S: Spike
ACE 2: Angiotensin-converting enzyme 2
RAS: Renin angiotensin system
ACE: Angiotensin-converting enzyme
PAI-1: Plasminogen activator inhibitor 1
VWF: Von Willebrand factor
CC: Coagulation consumption
CIC: COVID-19 induced coagulopathy
Poly P: Polyphosphates
